# Predicting cardiometabolic multimorbidity trajectory in middle-aged and older Chinese adults: insights from the cohort study on global ageing and adult health

**DOI:** 10.3389/fmed.2026.1890290

**Published:** 2026-07-08

**Authors:** Linlin Xie, Fei Wu, Huishan Li, Zhigang Wu, Ziyi Liang, Keqing Liang, Jianxiong Hu, Zishan Huang, Yizhen Yao, Jiamei Zeng, Jie Wan, Zongzhi Zhang, Tao Liu, Wenjun Ma, Fan Wu, Guanhao He

**Affiliations:** 1 Department of Public Health and Prevention Medicine, School of Medicine, Jinan University, Guangzhou, China; 2 School of Public Health, Fudan University, Shanghai, China

**Keywords:** cardiometabolic multimorbidity, risk factors, prediction model, cohort study, middle-aged and older Chinese adults

## Abstract

**Background:**

The ability to predict cardiometabolic multimorbidity (CMM) could significantly facilitate the identification of and intervention for middle-aged and older Chinese adults at risk. This study aimed to develop a prediction model for CMM progression trajectories based on multidimensional risk factors using an ensemble machine learning approach.

**Methods:**

Data from 4,518 participants were obtained from the World Health Organization’s Study on Global AGEing and Adult Health (SAGE) in China, covering the period from 2007 to 2019. Information on the incidence of cardiometabolic diseases (CMDs) was collected via self-reported surveys. CMM was defined as the presence of at least two CMDs, including hypertension, diabetes, angina, stroke, and obesity. A multi-state model was used to examine the influence of multidimensional factors on the transition from health to a single CMD and subsequently to CMM. Predictive models for these transitions were then developed.

**Results:**

During follow-up, 52.19% of initially healthy individuals developed one cardiometabolic disease (CMD), among whom 15.61% progressed to CMM. Female, low GDP per capita, unhealthy behaviors, elevated PM_2.5_ concentrations, low humidity, and low temperatures were identified as shared risk factors across the progression from health to CMD and from CMD to CMM. Moreover, in the health-to-CMD transition, older age, low educational level, and physical fitness impairment emerged as independent risk factors. Distinctively, in the CMD-to-CMM transition, IC impairment was identified as an independent influencing factor. Using these key predictors, a stacking ensemble model incorporating five machine learning algorithms was developed, and the model demonstrated area under the curve (AUC) values of 0.89 (95% CI: 0.88–0.91) for predicting the transition from health to CMD, and 0.76 (95% CI: 0.72–0.82) for predicting progression from CMD to CMM.

**Conclusion:**

In conclusion, this study demonstrates that a stacking ensemble model based on multidimensional factors can effectively predict the progression of CMM. Our study not only identified distinct risk factors for different transitional stages but also highlighted the potential of machine learning to improve early risk stratification and inform targeted interventions for preventing CMM in the aging.

## Introduction

Multimorbidity is a critical global health challenge since it reduces life quality and greater use of health-care resources (1). In China, rapid population aging has resulted in a significant burden of multimorbidity (2), and a study reported that 68.50% individuals aged 75 years and older suffered from multimorbidity (3). Cardiometabolic multimorbidity (CMM), defined as the simultaneous presence of at least two cardiometabolic diseases (CMDs), is one of the most frequently observed and replicable multimorbidity profiles (4, 5). The prevalence of CMM in Chinese adults (≥ 45 years) is 24.5% (6), which is higher than other frequently identified multimorbidity pattern, such as degenerative multimorbidity, which stands at 14.58% (7). Compared with participants without multimorbidity, those with CMM demonstrated twofold increased risk of mortality (8), while experiencing a life expectancy reduction of up to 15 years (9).

Identifying the key factors of CMM facilitates targeted prevention and control strategies. A prior prospective cohort study has identified unhealthy lifestyles, such as smoking, higher alcohol consumption and lower physical activity were associated with multimorbidity incidence in Europe elderly (10). A large prospective cohort study from the UK revealed that air pollutants play a hazardous role in the progression trajectory of CMM (11). However, previous studies often concentrate on merely one or two dimensions of risk factors, lacking a comprehensive, multidimensional framework for CMM. This fragmented focus inevitably obscures the stage-specific nature of various determinants across different phases of CMM progression. Since the factors associated with the onset of a first CMD may differ from those related to progression toward CMM, identifying stage-specific determinants may help inform targeted prevention strategies. However, evidence on such heterogeneity across different stages of CMM progression remains limited. Furthermore, those studies assumed that the risk factors remained unchanged throughout the entire follow-up period, which may lead to bias (12, 13).

Prediction model based on machine learning (ML) serves a crucial role in the early detection and intervention of CMM (14). For instance, a U. S. study has confirmed that a warning system based on gradient boosted trees reduced mortality risk by 18% in hospitalized sepsis patients (15), while a doctor-led mobile health intervention based on the China-PAR model achieved a 6.3% reduction in 10-year risk of atherosclerotic cardiovascular diseases among rural residents ((16, 17)). In China, although notable advancements have been achieved in developing predictive models for a single CMD (18, 19), a significant gap remains in the development of prediction models on CMM trajectory. Furthermore, prior studies have mostly relied on single prediction model, which may exhibit instability in performance. In contrast, ensemble learning, which uses a meta-learner to integrate predictions from multiple models, frequently achieving superior performance compared to individual model (20, 21).

This study used data from the World Health Organization Study on Global Ageing and Adult Health (WHO SAGE) China to develop prediction model on the trajectory of CMM based on multidimensional risk factors. Our findings are helpful to improve early risk stratification and inform targeted interventions for preventing CMM in rapid aging China.

## Methods

### Study design and participants

All participants were selected from WHO SAGE, a prospective cohort study in six countries (China, Ghana, India, Mexico, Russia, and South Africa) (22). The Chinese part of the SAGE was conducted in seven provinces (Jilin, Zhejiang, Shandong, Guangdong, Hubei, Shaanxi, and Yunnan) and one municipality (Shanghai), with one rural and one urban site selected in each region. People aged 50 years and over were selected as the study participants using a stratified multistage random cluster sampling (23).

This study utilized a dynamic cohort design based on three waves of the SAGE China study: Wave 1 (2007–2010), Wave 2 (2014–2015), and Wave 3 (2018–2019). To make full use of the longitudinal data, we included participants who completed at least two surveys. The analytic sample comprised participants enrolled in Wave 1 who were followed up in Wave 2 and/or Wave 3, as well as those who were newly recruited in Wave 2 and subsequently followed up in Wave 3. Integrating these groups maximizes the total sample size available for estimating transition probabilities across the progression of CMM. From an initial pool of 18,673 participants across all waves, we applied the following exclusion criteria: (1) failed to complete at least one subsequent follow-up; (2) aged <50 years at their respective baseline; (3) had missing data on key variables; (4) had prevalent CMDs at baseline. Ultimately, a total of 4,518 participants were included in the final analysis (Supplementary Figure S1).

### Identification of chronic diseases

In this study, CMM was defined as the coexistence of at least two of the following five conditions: obesity, hypertension, diabetes, angina, stroke. The selection of these five specific diseases is empirically supported by prior clustering evidence utilizing the WHO SAGE database and is consistent with previous epidemiological studies of CMM (5, 24). Obesity is specifically identified when an individual’s body mass index (BMI) is equal to or greater than 30, while the status of other chronic disease was assessed by two questions. For instance, “Have you ever been diagnosed with hypertension during last 12 months?” and “Have you been taking any medications or other treatments for this hypertension last 12 months?” If either of the two questions in the questionnaire has an answer of “Yes,” then we will define the respondent as a hypertension case (25).

### Data collection

Baseline data were collected through face-to-face interviews with all enrolled participants. During follow-up investigations, participants also underwent similar face-to-face interviews to update information on morbidity. The study was approved by the Ethics Committee of the Chinese Centre for Disease Control and Prevention (approval number: 200601), and by the Shanghai Municipal Center for Disease Control and Prevention (approval number: 2014-8, 2018-1). Informed consent was obtained from each participant before the investigation.

### Risk factors

According to prior studies (11, 16, 17, 26, 27), this study on multimorbidity based on five dimensions: socio-demographic characteristics, behavioral factors, physiological indicators, IC and environmental exposure. Socio-demographic dimensions incorporate sex, age, marital status, education, Gross Domestic Product (GDP) per capita. Behavioral factors integrate smoking status, drinking status, physical activity, social engagement, and vegetable intake. Physiological indicators include grip strength, pulmonary function, pulse and blood pressure. Intrinsic capacity (IC) focuses locomotion, vitality, cognition, depression, sensory. We constructed unhealthy behavior scores, physical fitness impairment scores and IC impairment scores with different modifiable factors, respectively. Participants were assigned 1 point for each factor against with guidelines and recommendations, with higher scores indicating worse adherence to the corresponding domain. For instance, behavior factors domain included five conventional factors (smoking status, drinking status, physical activity, social engagement and vegetable intake), and the participants who reported smoke, drink, low physical activity, insufficient social engagement and inadequate vegetable intake accumulated up to 5 points in “unhealthy behavior” variable. Details of the definitions of risk factors were showed in Supplementary Table S1, and Supplementary Tables S2–S4 outline the specific scoring criteria for unhealthy behavior, physical fitness impairment and IC impairment.

For environmental exposure, we include ambient fine particulate matter less than 2.5 (PM_2.5_), average temperature and relative humidity. PM_2.5_ data were obtained from the high-resolution (1 km × 1 km) daily dataset provided by the National Earth System Science Data Center’s Shared Service Platform^1^. Validation studies of this dataset demonstrate excellent agreement with ground monitoring stations, showing a coefficient of determination (*R*^2^) of 0.92 and root mean square error (RMSE) of 10.76 μg/m^3^. The meteorological data include the annual average temperature and relative humidity are derived from the Fifth Generation Atmospheric Reanalysis Dataset (ECMWF ReAnalysis 5, ERA5) of the European Centre for Medium-Range Weather Forecasts. The ERA5 dataset provides a latitude-longitude network with a spatial resolution of 0.1° × 0.1° (equivalent to approximately 9 km × 9 km in the mid-latitude region). For each participant, we extracted daily environmental data based on geographic coordinates and estimated average exposures of PM_2.5_ concentrations, temperature and relative humidity values from the 1 year preceding each investigation to the survey date.

Demographic characteristics were assessed at baseline, whereas other variables were updated at each wave. This time-updated approach captures the dynamic status of participants during each transition interval across the entire follow-up period.

Missing values in variables were imputed using multiple imputation by chained equations (MICE), ensuring the robustness of the analyses.

### Statistical analysis

#### Multi-state model

In the study, two transition stages of multimorbidity were established: (1) Health to CMD; (2) CMD to CMM. For each participant, survival time was calculated from baseline until either the occurrence of the respective transition event or censoring, whichever occurred first. The exact date of a transition event was determined by the survey date where the diagnostic criteria were first met. Participants who remained event-free at the final follow-up or were lost to follow-up were defined as censoring. We further adopted the unidirectional multi-state model with Markov proportional hazards, an extension of the conventional Cox model, to simultaneously investigate the impact of multidimensional factors on different phases of disease progression (i.e., Health, to CMD, to CMM). The formula was presented as follow:

$$\lambda_{\mathrm{ij}}(t\;|Z)=\lambda_{\mathrm{ij},0}(t)\exp(\beta_{\mathrm{ij}}^{T}Z)$$

$$\lambda_{\mathrm{ih}}(t)=\lim_{△t\to 0}\frac{P_{\mathrm{ih}}(t,t+△t)=j|X(t)=i}{△t}$$

$$P_{\mathrm{ij}}(s,t)=\text{Prob}(X(t)=j\;|\;X(s)=i)$$

In this multi-state model, $\lambda_{\mathrm{ij}}(t\;|Z)$ represents the instantaneous transition hazard from state i to state j at time t, given a set of covariates Z, while $\lambda_{\mathrm{ij},0}(t)$ denotes the baseline hazard for the same transition when all covariates are set to zero. Here, Z represents the covariate vector, and $\beta_{\mathrm{ij}}$ corresponds to the regression coefficients specific to the transition of i to j. A Markov proportional hazards model was incorporated to evaluate the effects of these covariates on different transition pathways simultaneously.

#### Machine learning algorithms

Candidate predictors were primarily selected based on previous epidemiological evidence (11, 16, 17, 26). To further refine the feature set, we incorporated all potential risk factors (*p* < 0.05) identified in the progression trajectory of CMM into the prediction models. The dataset was partitioned into training (70%) and testing (30%) subsets using random sampling to preserve outcome distribution across both sets. This approach ensured the training set would be used for model development while maintaining an independent testing set for unbiased performance evaluation.

The stacking ensemble model is a ML technique that combines multiple base models to improve predictive performance. The stacking framework operates through a hierarchical structure comprising two key components: base models and a meta-model. In stacking, meta-models are trained to learn how to best combine the predictions of the base models. During the first stage, each base-model classifier is independently trained on the initial training dataset. These base models then generate predictions that serve as input features for the subsequent meta-learner. First, we used five algorithms, eXtreme Gradient Boosting (XGBoost), Random Forest (RF), Logistic Regression (LR), Support Vector Machine (SVM), and Neural Network (NN) as the base models, which were widely used in the study of diseases prediction (27, 28). To ensure robust generalization and prevent overfitting, we utilized a 5-fold cross-validation (CV) strategy on the training dataset to generate “out-of-fold” predictions. Specifically, the training set was divided into five subsets; each base model was trained on four subsets and made predictions on the remaining “held-out” subset. This process was rotated until every observation in the training set had a corresponding meta-feature generated by each base model. Hyperparameters for all base models were optimized via grid search within the internal CV loops. Second, logistic regression was chosen as a meta-model, which effectively aggregated the predictions from base models to improve overall predictive accuracy (29). In this study, the aim of using an integrated ML model is to take full advantage of different classes of the five different ML algorithms and improve the overall performance of the prediction model. Finally, the performance of the fully trained stacking ensemble was evaluated on a completely independent test set (30% of the total sample) using AUC-ROC, sensitivity, specificity, accuracy, Brier score, calibration slope and intercept.

To visually interpret the global predictive contribution of multi-dimensional features to the ultimate decisions of the integrated framework, a weighted ensemble SHapley Additive exPlanations (SHAP) approach was further developed. The empirical regression coefficients of each base learner were extracted from the trained meta-learner and normalized to serve as weights. Subsequently, individual SHAP matrices for the five base learners (XGBoost, Random Forest, Logistic Regression, Support Vector Machine, and Neural Network) were first computed on the independent 30% testing dataset via the fastshap algorithm. The overall stacking SHAP values were then derived via a weighted linear combination of the individual base learners’ SHAP matrices ( $\mathrm{SHA}P_{\text{stacking}}=\sum \text{Weig}ht_{i}\times \mathrm{SHA}P_{i}$ ). The final global feature importance was ranked and visualized using the mean absolute SHAP values for the health-to-CMD and CMD-to-CMM transitional stages, respectively.

Furthermore, multicollinearity among all candidate predictors from multiple domains was rigorously assessed using the Variance Inflation Factor (VIF). All candidate variables exhibited VIF values well below the conservative threshold of 5 (with all VIFs < 1.6), demonstrating the absence of severe multicollinearity in our multi-dimensional framework (Supplementary Figure S2).

#### Sensitivity analysis

To assess the robustness of our primary findings, we conducted several sensitivity analyses: (i) adjusting the exposure duration (2 and 3 years) of the ambient PM_2.5_ concentrations, temperature and relative humidity; (ii) employing traditional Cox proportional hazards models to analyze each distinct state transition individually. (iii) Excluding respondents with other pre-existing chronic conditions at baseline and re-establishing the prediction models to observe the changes in there predictive performance. (iv) Re-fitting the multi-state model using a random 70% subsample to ensure that the feature selection remained stable. (v) Redefining obesity using the population-specific cut-off for Chinese temporal traits (BMI ≥ 28 kg/m^2^) and re-fitting both the multi-state trajectory models and the machine learning predictive frameworks to evaluate the robustness.

Data analysis was performed with the statistical software package R (v4.4.1). The R packages survival, msm, mstate were utilized for statistical analysis, and a *p*-value < 0.05 was considered statistically significant.

## Results

### Descriptive results

The baseline characteristics of the 4,518 study participants are summarized in Table 1. The demographic breakdown revealed that 52.43% (*n* = 2,369) were male, 45.79% (*n* = 2,069) were aged ≥ 60 years, 87.78% (*n* = 3,966) were married, and 68.39% (*n* = 3,090) had at least a primary education. Half of the cohort (53.61%, *n* = 2,422) lived in low-GDP counties. Mean values for IC impairment, unhealthy behavior and physical fitness impairment scores were 1.72 (±1.34), 2.01 (±1.03), and 1.33 (±0.75), respectively. The average annual environmental exposures were 54.32 ± 13.03 μg/m^3^ for PM_2.5_, 15.40 ± 4.05 °C for temperature, and 71.41 ± 5.23% for relative humidity. Compared to the baseline healthy status, those progressing to CMD and CMM had higher aging and unmarried proportion, as well as increased level of IC impairment, unhealthy behavior and physical fitness impairment.

**Table 1:** Basic information of the study participants during CMM progression.

Group	Number (%) / Mean ± sd		
	Health (Baseline)	CMD	CMM
Total	4,518 (100.00%)	2,358 (52.19%)	368 (15.61%)
Sex			
Male	2,369 (52.43%)	1,254 (53.18%)	183 (49.73%)
Female	2,149 (47.57%)	1,104 (46.82%)	185 (50.27%)
Age (year)			
<60	2,449 (54.21%)	411 (17.43%)	48 (13.04%)
≥60	2,069 (45.79%)	1,947 (82.57%)	320 (86.96%)
Marital status			
Married	3,966 (87.78%)	2,031 (86.13%)	313 (85.05%)
Unmarried	552 (12.22%)	327 (13.87%)	55 (14.95%)
Education			
Less than primary	1,428 (31.61%)	822 (34.86%)	114 (30.98%)
Primary and above	3,090 (68.39%)	1,536 (65.14%)	254 (69.02%)
GDP per capita (Yuan)			
High	2,096 (46.39%)	1,085 (46.01%)	206 (55.98%)
Low	2,422 (53.61%)	1,273 (53.99%)	162 (44.02%)
**IC impairment**	1.72 ± 1.34	2.06 ± 1.45	2.47 ± 1.44
**Unhealthy behavior**	2.01 ± 1.03	2.72 ± 1.10	2.74 ± 1.14
**Physical fitness impairment**	1.33 ± 0.75	1.50 ± 0.71	1.59 ± 0.77

The IC impairment was calculated based on mobility, vitality, cognition, depression and sensory (range 0–5). Unhealthy behavioral was calculated based on smoking, drinking, physical activity, social status and vegetable intake (range 0–5). Physical fitness impairment was calculated based on grip strength, lung capacity, pulse rate and prehypertension (range 0–4). Details are provided in Supplementary Tables S2–S4.

### The progression trajectories of CMM

**Figure 1:**
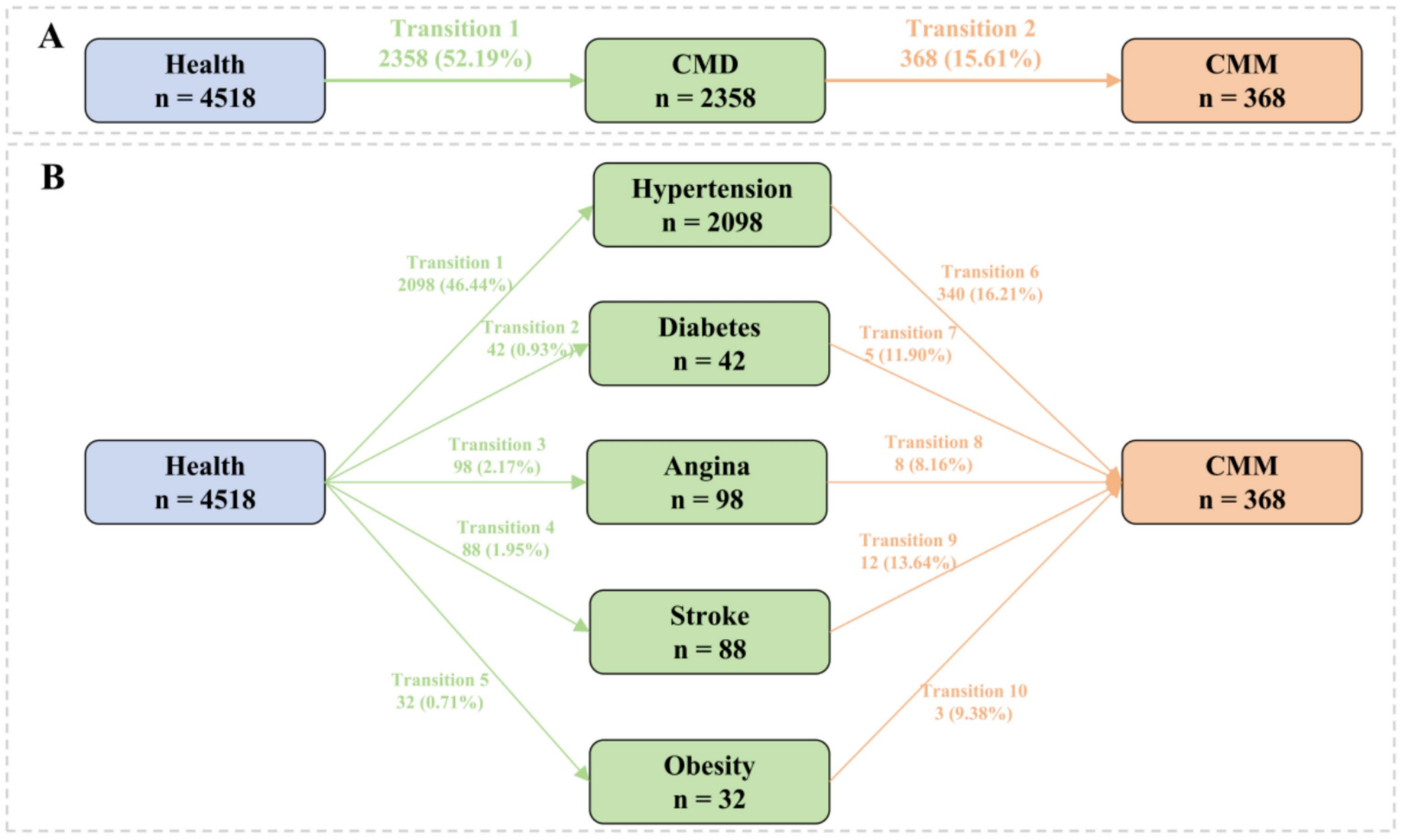
Status transition of CMD. **(A)** The numbers (percentages) of participants from health to all CMD and CMM. **(B)** The numbers (percentages) of participants from health to specific CMD and from specific CMD to CMM.

During a median follow-up of 8.72 years, 52.19% (2,385) of initially healthy people developed a single CMD, of whom 15.61% (368) progressed to CMM. From health to CMD, 2,098 (46.44%) of CMDs were hypertension, 42 (0.93%) were diabetes, 98 (2.17%) were angina, 88 (1.95%) were stroke, and 32 (0.71%) were obesity. Compared with participants with diabetes, angina, stroke and obesity, participants with hypertension, were more likely to develop CMM. Stage-specific transitions are detailed in Figure 1.

### Risk factors of CMM progression trajectories

**Figure 2:**
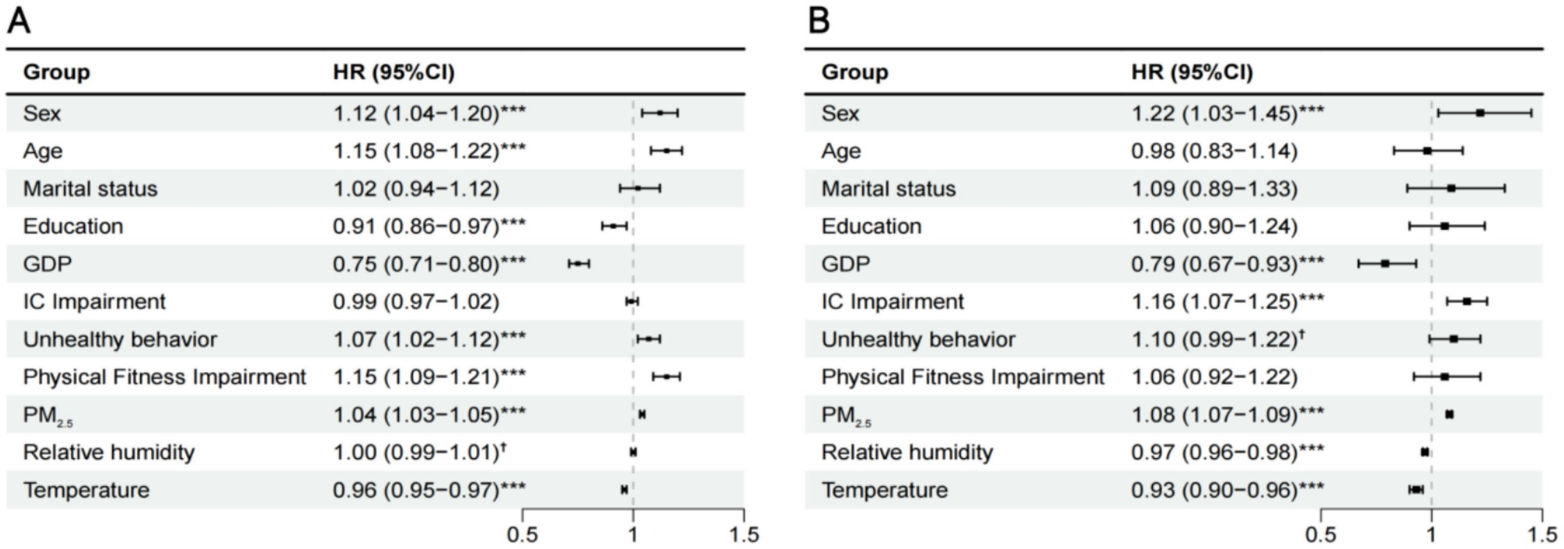
The muti-state model analysis of multidimensional risk factors. **(A)** Hazard ratios (HRs) and 95% confidence intervals (CIs) from health to CMD. **(B)** Hazard ratios (HRs) and 95% confidence intervals (CIs) from CMD to CMM. CMD: cardiometabolic disease (hypertension, diabetes, angina, stroke or obesity); CMM, cardiometabolic multimorbidity: a status with at least two CMDs. ***: Significant interaction effect at *p* < 0.001, **: Significant interaction effect at *p* < 0.01, *: Significant interaction effect at *p* < 0.05, †: Marginal effect at *p* < 0.1. Ref: Sex (male), Age (<60 years), Education (less than primary), GDP per capita (Low), PM_2.5_ (per 1 μg/m3).

Multi-state analyses showed that female (HR = 1.12, 95% CI: 1.04–1.20), older age (HR = 1.15, 95% CI: 1.08–1.22), unhealthy behavior (HR = 1.07, 95% CI: 1.02–1.12), physical fitness impairment (HR = 1.15, 95% CI: 1.09–1.21) and elevated PM_2.5_ concentrations (HR = 1.04, 95% CI: 1.03–1.05) significantly increased the risk from health to develop CMD, while higher education levels (HR = 0.91, 95% CI: 0.86–0.97), higher GDP per capita (HR = 0.75, 95% CI: 0.71–0.80), and warmer ambient temperature (HR = 0.96, 95% CI: 0.95–0.97) demonstrated significant protective effects (Figure 2A). Female (HR = 1.22, 95% CI: 1.03–1.45), IC impairment (HR = 1.16, 95% CI: 1.07–1.25), elevated PM_2.5_ concentrations (HR = 1.08, 95% CI: 1.07–1.09) were significantly associated with an increased risk from CMD to CMM, while higher relative humidity (HR = 0.97, 95% CI: 0.96–0.98) and warmer temperature (HR = 0.93, 95%CI: 0.90–0.96) was protective factors (Figure 2B).

### Prediction models for CMM multimorbidity trajectories

**Figure 3:**
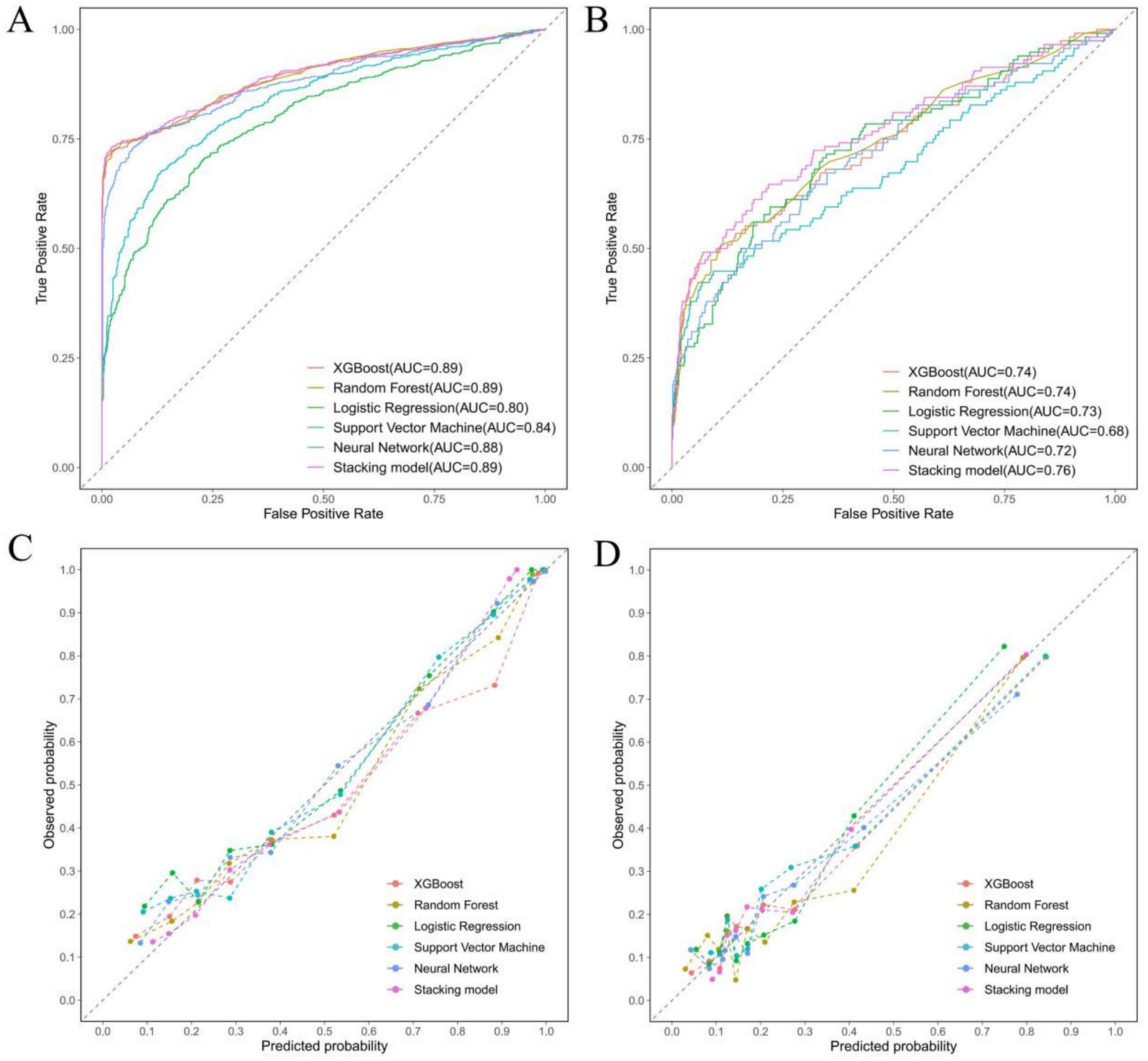
ROC curve and calibration curve of the Machine Learning prediction models. **(A)** ROC curve: Health to CMD; **(B)** ROC curve: CMD to CMM; **(C)** Calibration curve: Health to CMD; **(D)** Calibration curve: CMD to CMM.

Based on the key risk factors abovementioned, we first developed prediction models of CMM trajectories using five algorithms: XGBoost, RF, LR, SVM, and NN. All models exhibited predictive capability across transitions from healthy status to CMD, with better performance for XGBoost (AUC = 0.89, 95%CI: 0.88–0.91) and RF (AUC = 0.89, 95%CI: 0.87–0.90), followed by NN (AUC = 0.88, 95% CI: 0.86–0.90), SVM (AUC = 0.84, 95% CI: 0.82–0.85) and LR (AUC = 0.80, 95% CI: 0.78–0.82). For CMD to CMM, models showed moderate performance for XGBoost (AUC = 0.74, 95% CI: 0.68–0.79), RF (AUC = 0.74, 95% CI: 0.69–0.80), LR (AUC = 0.73, 95% CI: 0.68–0.78) and NN (AUC = 0.72, 95% CI: 0.66–0.77) (Figure 3).

We further developed a stacking ensemble model by integrating the five algorithms. The models demonstrated robust diagnostic performance for trajectory from health to CMD and from CMD to CMM, achieving an AUC of 0.89 (95%CI: 0.88–0.91) and 0.76 (95%CI: 0.72–0.82), respectively. Moreover, the ensemble learning models demonstrated well overall predictive performance. The prediction model for transitions from health to CMD showed high sensitivity (0.74), specificity (0.98), and accuracy (0.85), while the model for CMD to CMM progression attained corresponding values of 0.65, 0.78 and 0.76, respectively. Additionally, calibration performance was satisfactory for both transitions. For the Health-to-CMD transition, the Stacking model achieved a Brier score of 0.12, a calibration slope of 0.93, and an intercept of 0.02. For the CMD-to-CMM transition, the corresponding values were 0.12, 1.05, and −0.02, respectively. The other detailed information on the stacking ensemble model is presented in Supplementary Figures S3, S4 and Supplementary Tables S5, S6.

**Figure 4:**
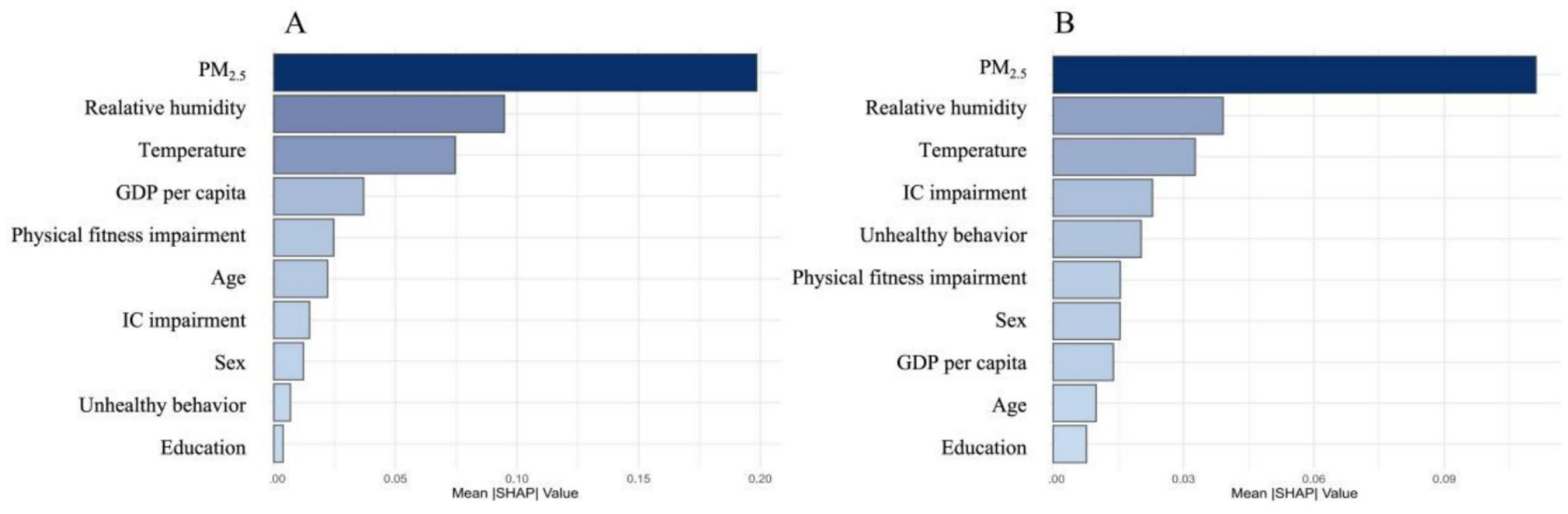
Global feature importance of the predictive models based on the weighted Stacking ensemble SHAP approach. **(A)** Global feature importance ranked by the mean absolute overall SHAP values from health to CMD. **(B)** Global feature importance ranked by the mean absolute overall SHAP values from CMD to CMM.

To further interpret the predictive driving mechanism of the Stacking framework, global feature importance was evaluated using the mean SHAP values. For the transition from health to CMD, environmental factors along with GDP per capita and age emerged as the top 5 predictive risk factors (Figure 4A). In contrast, for the subsequent progression from CMD to CMM, while environmental exposures remained prominent, IC impairment and unhealthy behavior escalated into the top 5 key drivers (Figure 4B).

### Sensitivity analyses

The robustness of our findings was confirmed through a series of sensitivity analyses. First, to assess the robustness of our exposure definitions, we extended the period for calculating moving averages of ambient PM_2.5_, temperature, and relative humidity from 1–2 years and 3 years. The associations observed in the multi-state model remained virtually unchanged (Supplementary Tables S7, S8). Second, to verify the consistency of the risk factors identified by the multi-state model, we performed a traditional Cox regression analysis for each transition (Health to CMD, CMD to CMM) separately. The results were consistent with those from multi-state model, confirming the reliability of our core findings (Supplementary Table S9). Third, to ensure that our results were not confounded by other diseases, we repeated both the multi-state modeling and the machine learning prediction analyses in a sub-cohort excluding individuals. In this analysis, we excluded individuals who had pre-existing chronic conditions at baseline, including any of the five pre-defined CMDs or other chronic diseases (Supplementary Table S10). The identified risk factors (Supplementary Table S11) and the performance of the prediction models (Supplementary Tables S12, S13) in this healthy sub-cohort were also performed well. Additionally, to address concerns regarding potential data leakage in the prediction models, we re-fitted the multi-state model using a random 70% subsample. The significant predictors in this subsample were highly consistent with those identified in the full population, confirming that our feature selection was robust and independent of the test set (Supplementary Table S14). Finally, to address potential diagnostic variations in body composition metrics, we reperformed the complete workflow using the Chinese population-specific obesity threshold (BMI ≥ 28 kg/m^2^). Under this native criteria framework, both the hazard ratios in the multi-state trajectories and the predictive evaluation indicators of the Stacking framework demonstrated exceptional stability and closely matched the primary analysis results (Supplementary Tables S15–S17).

## Discussion

In this national prospective cohort study in China, this study identified both common and distinct risk factors associated with the progression from health to CMD, from CMD to CMM. Notably, female, low GDP per capita, unhealthy behaviors, elevated PM_2.5_ concentrations, low humidity, and low temperatures were identified as shared risk factors across the progression from health to CMD and from CMD to CMM. Moreover, in the health-to-CMD transition, older age, low educational level, and physical fitness impairment emerged as independent risk factors. Distinctively, in the CMD-to-CMM transition, IC impairment was identified as an independent risk factor. The results highlight the complexity of disease progression pathways, emphasizing the need for stage-specific interventions. Based on the list of risk factors, we constructed two stacking ensemble models with good prediction performance by integrating five machine learning algorithms models, which achieved good prediction performance from health to CMD, further to CMM. Our finding could provide precise and feasible tools for early risk prediction and early warning of CMM.

Our study identified several shared risk factors that were consistently associated with disease progression, from health to CMD and onward to CMM. These include socio-demographic characteristics, lifestyle patterns and environmental exposures, creating a sustained pathogenic milieu across CMM stages. First, socio-demographic factors such as female and lower GDP per capita exert a dual-phase influence. The elevated risk of female might be attributable to post-menopausal hormonal shifts and vascular stiffness, which exacerbates cardiovascular risks (30). Moreover, lower GDP per capita may lead to fragmented healthcare systems and limited health-promoting resources, thereby possibly impeding effective chronic disease prevention and management (31). Second, modifiable behavioral factors, including smoking, excessive alcohol consumption, physical inactivity, poor diet, and limited social engagement, that were collectively related to progression from health to CMD and further to CMM, consistent with Western longitudinal studies (32–34). Specifically, tobacco use and chronic alcohol use promote systemic inflammation and endothelial dysfunction, while physical inactivity disrupts lipid metabolism (35, 36). Furthermore, inadequate vegetable intake deprives essential antioxidants, dietary fiber, and anti-inflammatory phytochemicals, thus affecting metabolic regulation and vascular health (37). Beyond these traditional risks, social isolation may induce chronic psychological distress, amplifying sympathetic activity, inflammation, and depressive symptoms, thereby accelerating disease progression (38, 39). Our study identified associations of both conventional and emerging lifestyle factors with CMM progression, suggesting emerging factors may aid in prevention, control and delay.

Beyond individual-level factors, our results underscored that environmental exposures are associated with the dynamics of multimorbidity. The consistent risk posed by PM_2.5_ across disease transitions, alongside the protective roles of warmer temperature and higher humidity. Fine particulate matter may acts as a pervasive biological stressor, promoting disease via pathways like autonomic imbalance and chronic inflammation (40–42). Conversely, cold and dry conditions appear to impose acute hemodynamic and rheological strain, precipitating events in vulnerable individuals (43, 44). Given that epidemiology has demonstrated that air purification and central heating have benefit on cardiovascular health (45, 46), our findings further encourage these adaption strategies against CMM.

Our study identified distinct risk factors at either the onset of the first chronic condition or its progression to multimorbidity. Older age, lower educational level and physical fitness impairment were associated with increased risk from health to CMD. As individuals age, they experience a gradual decline in physiological reserves across multiple organ systems, coupled with immunosenescence and progressive vascular aging, and probably promote the onset of vascular-related diseases (47, 48). Lower educational level may be associated with insufficient ability of individuals to obtain and understand health information, thereby affecting their health behaviors. In addition, our findings aligned with existing evidence indicating that physical fitness impairment is significant risk factors for CMDs (26, 49–51). For instance, grip strength reflects muscle health, and its decline is linked to elevated systemic inflammation and worsened insulin resistance (52). Similarly, impaired lung function necessitates increased cardiac output for oxygenation, imposing hemodynamic strain on the heart and vasculature (53). Excessive pulse and blood pressure variability induce hemodynamic stress that can damage end-organs such as the brain and heart, further raising CMD risk (54, 55).

Notably, we identified impaired IC as a significant predictor of progression from CMD to CMM, underscoring its considerable role in the transition toward greater multimorbidity. This is consistent with recent studies linking IC deficits to higher cardiometabolic incidence (56, 57) and functional decline over time (58, 59). IC encompasses five core domains, including locomotion, cognition, vitality, psychological state, and sensory function, which collectively reflecting the integration of multiple physiological systems. Its impairment may act through shared pathways such as chronic inflammation, neuroendocrine imbalance, and autonomic dysfunction (60, 61), not only as a marker but potentially tracking with multisystem dysregulation in individuals already burdened by an initial chronic condition, which suggesting that interventions aimed at preserving or restoring IC may interrupt the cascade toward additional multimorbidity.

This stage-specific divergence indicates that older age, lower education, and physical fitness impairment contribute more to the onset of the first chronic condition than to the later build-up of comorbidity, whereas IC impairment appears more relevant during the disease accumulation phase. Older age and lower educational attainment can be regarded as upstream determinants of disease onset (62–64). Aging is associated with the gradual accumulation of cardiometabolic risk, while individuals with higher education are generally more likely to access health information, adopt preventive behaviors, and seek timely medical care. Once a cardiometabolic disease is diagnosed, patients commonly receive regular medical follow-ups and health advice. This ongoing care helps close the gap in disease awareness that often exists between age and educational groups, and it shifts the main drivers of further disease accumulation toward disease management and treatment adherence, rather than leaving them dependent on chronological age or educational attainment alone. Similarly, physical fitness impairment may reflect early physiological vulnerability. Following the onset of a chronic disease, progression toward multimorbidity may be influenced more strongly by broader functional decline, thereby reducing the relative contribution of these early physiological indicators(65). In contrast, impaired IC may reflect reduced physiological reserve and resilience across multiple functional domains(66, 67). Among individuals who already have a cardiometabolic disease, lower IC may indicate a diminished capacity to cope with disease-related stressors, making them more susceptible to the development of additional chronic conditions.

To our knowledge, this study is the first to apply prediction models to integrate multidimensional factors to construct prediction models spanning the progression from health to CMD, subsequently to CMM, respectively. In China, a ten metabolites-based algorithm demonstrated robust predictive performance for type 2 diabetes (T2D) onset (AUROC = 0.89, 95%CI: 0.86–0.93) (68), which was comparable to our health-to-CMD prediction model (AUC = 0.89, 95%CI: 0.88–0.91). While prior research in China has established highly effective prediction models for individual CMD, studies addressing CMM prediction remain scare. Notably, our CMD-to-CMM prediction model (AUC = 0.76, 95%CI: 0.72–0.82) exhibited performance equivalent to a Cox regression prediction model for CMM events based on UK Biobank (AUC = 0.72, 95%CI: 0.71–0.73) (69). Furthermore, we constructed a stacking ensemble model integrating XGBoost, LR, RF, SVM and NN for CMM trajectories. Consistent with previous study (21), the stacking model demonstrated superior predictive performance compared to other single models, which could improve accuracy by reducing outliers to highlight its potential as a reliable approach for disease prediction (70). Our prediction models could provide a certain support for the early identification and intervention of CMM.

This study underscores key public health implications for CMM progression. It identifies both shared risk factors (e.g., unhealthy behaviors, environmental exposures) acting across stages from CMD and to CMM, and stage-specific factors (e.g., physical fitness in initial onset, intrinsic capacity in progression to multimorbidity). This highlights the necessity for integrated interventions targeting common risks throughout the disease progression, complemented by stage-tailored strategies addressing distinct factors at each transition. Furthermore, the developed stacking ensemble prediction models enable early risk prediction and warning for both transitions. Implementing such models facilitates timely, precise interventions, which is crucial for delaying or preventing the progression to multimorbidity.

### Strengths and limitations

Our study presents several strengths. First, we considered multidimensional factors rather than relying on a single dimension, enabling a comprehensive characterization of risk factors influencing the progression trajectory of CMM. Second, this is the first use of ensemble model to predict the progression of CMM in a Chinese population, contrasting with conventional single-model prediction frameworks. Several limitations should also be acknowledged. First, the cohort had a limited number of follow-up visits, which may limit the capacity to capture long-term CMM progression dynamics. Second, ambient environmental exposure in our study were estimated based on participants’ residential addresses without accounting for daily activities and location, potentially introducing exposure misclassification. Third, some environmental pollutants were not available prior to 2013 (e.g., SO_2_, NO_2_, CO). Consequently, only PM_2.5_ was taken into consideration. Additionally, certain conditions such as obesity may be reversible; however, the multi-state model assumed irreversible transitions, which may not fully capture instances of health recovery. Finally, the shorter follow-up for Wave 2 recruits might limit the observation of disease progression events within the study period.

## Conclusion

In conclusion, this prospective cohort of middle-aged and older Chinese adults revealed that CMM progression is driven by both shared risk factors and stage-specific factors. We developed good-performance prediction models for both the health-to-CMD and CMD-to-CMM transitions. These findings support implementing integrated public health interventions targeting common risks, alongside stage-targeted strategies enabled by early prediction, to effectively delay or prevent CMM.

## Data Availability

The data analyzed in this study is subject to the following licenses/restrictions: the datasets used and analyzed during the current study are available from the corresponding author on reasonable request. Requests to access these datasets should be directed to heguanh1991@163.com (GH).

## Footnotes

1
http://www.geodata.cn/data/index.html
