# Supplementary material for: Predicting cardiometabolic multimorbidity trajectory in middle-aged and older Chinese adults: insights from the cohort study on global ageing and adult health
Source: Front Med (Lausanne). 2026 Jul 8;13:1890290. doi: 10.3389/fmed.2026.1890290 (PMC13388161; doi:10.3389/fmed.2026.1890290)
Supplement: Supplementary file 1 [file Data_Sheet_1.pdf]

Predicting cardiometabolic multimorbidity trajectory in middle-aged and older Chinese adults: insights from the cohort study on global AGEing and Adult Health (SAGE)

Supplemental Material

Author: Linlin Xie[1+], Fei Wu[2+], Huishan Li[1+], Zhigang Wu[1], Ziyi Liang[1], Keqing Liang[1], Jianxiong Hu[1], Zishan Huang[1], Yizhen Yao[1], Jiamei Zeng[1], Jie Wan[1], Zongzhi Zhang[1], Tao Liu[1], Wenjun Ma[1*], Fan Wu[2**], Guanhao He[1***].

Affiliations:

1. Department of Public Health and Prevention Medicine, School of Medicine, Jinan University, Guangzhou, 510632, China.

2. School of Public Health, Fudan University, Shanghai, 200032, China.

* Corresponding author. Department of Public Health and Prevention Medicine, School of Medicine, Jinan University, No.601, Huangpu Road, Tianhe District, Guangzhou 510632, China.

** Corresponding author. Department of Epidemiology, School of Public Health, Fudan University, No.130 Dongan Road, 200032 Shanghai, China.

*** Corresponding author. Department of Public Health and Prevention Medicine, School of Medicine, Jinan University, No.601, Huangpu Road, Tianhe District, Guangzhou 510632, China.

E-mail addresses: mawj@gdiph.org.cn (Ma Wenjun), wufan@scdc.sh.cn (Wu Fan), heguanh1991@163.com (He Guanhao).

[+] These authors contributed equally to this work.

Contents

Figure S1. The flow chart of the study population selection

Figure S2. Variance Inflation Factor (VIF) distribution across multi-dimensional candidate

Figure S3. The Decision curves of machine learning predictive models from health to CMD

Figure S4. The Decision curves of machine learning predictive models from CMD to CMM

Table S1. Definition of risk factors in the study [Total]

Table S2. Unhealthy behavior's definition in the study

Table S3. Physical fitness impairment's definition in the study

Table S4. Definitions of Intrinsic Capacity Measurement Items included in the study

Table S5. Indicators of predictive different models from Health to CMD

Table S6. Indicators of predictive different models from CMD to CMM

Table S7. Hazard ratios (HRs) and 95% confidence intervals (CIs) for each transition in CMM progression among participants with 2 year environmental exposures

Table S8. Hazard ratios (HRs) and 95% confidence intervals (CIs) for each transition in CMM progression among participants with 3 year environmental exposures

Table S9. Hazard ratios (HRs) and 95% confidence intervals (CIs) for each transition in CMM progression by Cox regression

Table S10. Basic information of the study participants in CMM progression without

baseline chronic conditions

Table S11. Hazard ratios (HRs) and 95% confidence intervals (CIs) for each transition in CMM progression among participants without baseline chronic conditions.

Table S12. Indicators of predictive different models from Health to CMD without baseline diseases

Table S13. Indicators of predictive different models from CMD to CMM without baseline diseases

Table S14. Hazard ratios (HRs) and 95% confidence intervals (CIs) for each transition in CMM progression among participants based on a 70% Random subsample

Table S15. Hazard ratios (HRs) and 95% confidence intervals (CIs) for each transition in CMM progression (obesity:BMI⩾28 kg/m^2)

Table S16. Indicators of predictive different models from Health to CMD (obesity:BMI⩾28 kg/m^2)

Table S17. Indicators of predictive different models from CMD to CMM (obesity:BMI⩾28 kg/m^2)

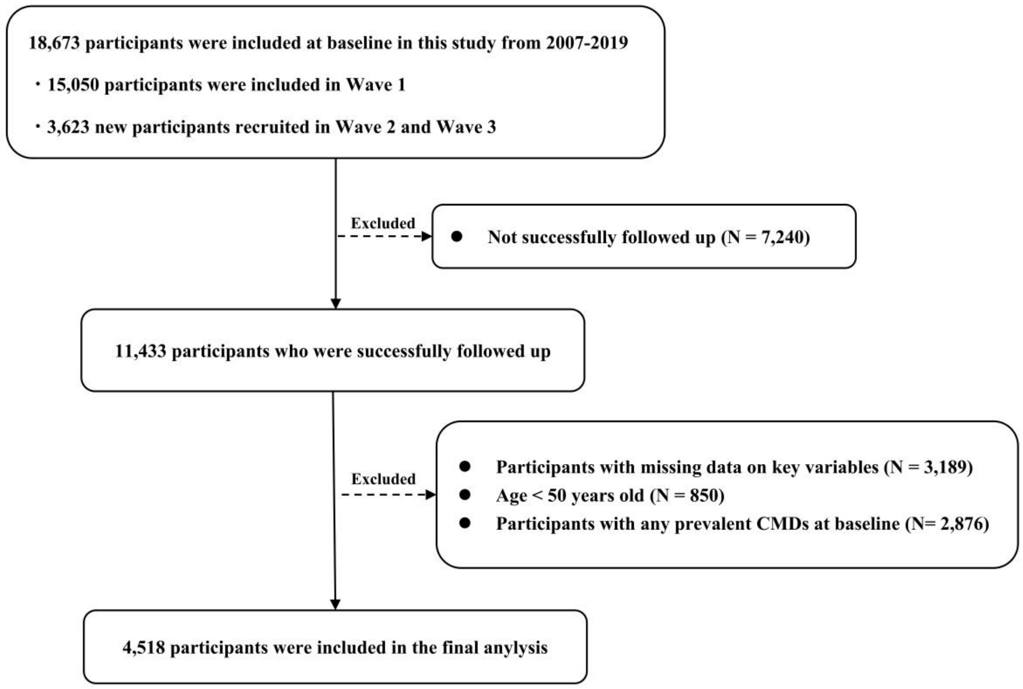

Figure S1. The flow chart of the study population selection.

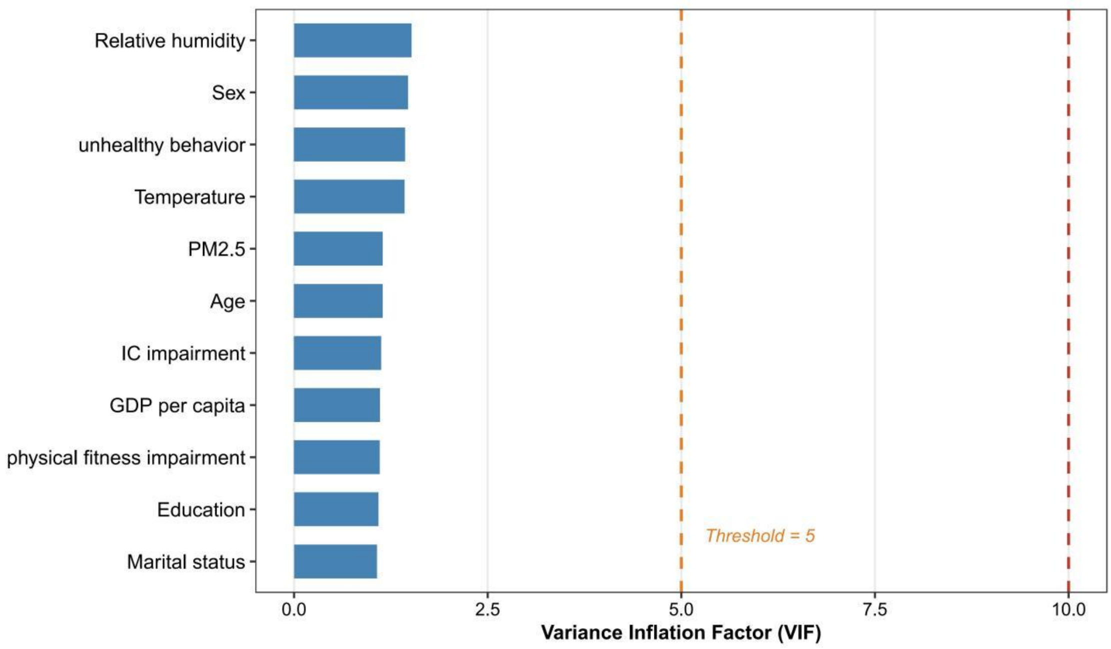

Figure S2. Variance Inflation Factor (VIF) distribution across multi-dimensional candidate

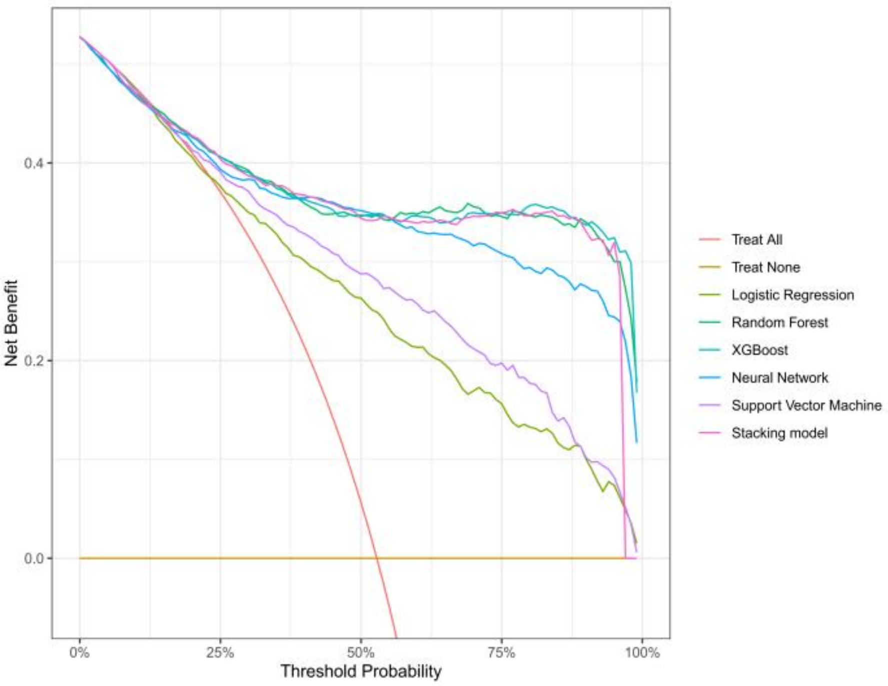

Figure S3. The Decision curves of machine learning predictive models from health to CMD.

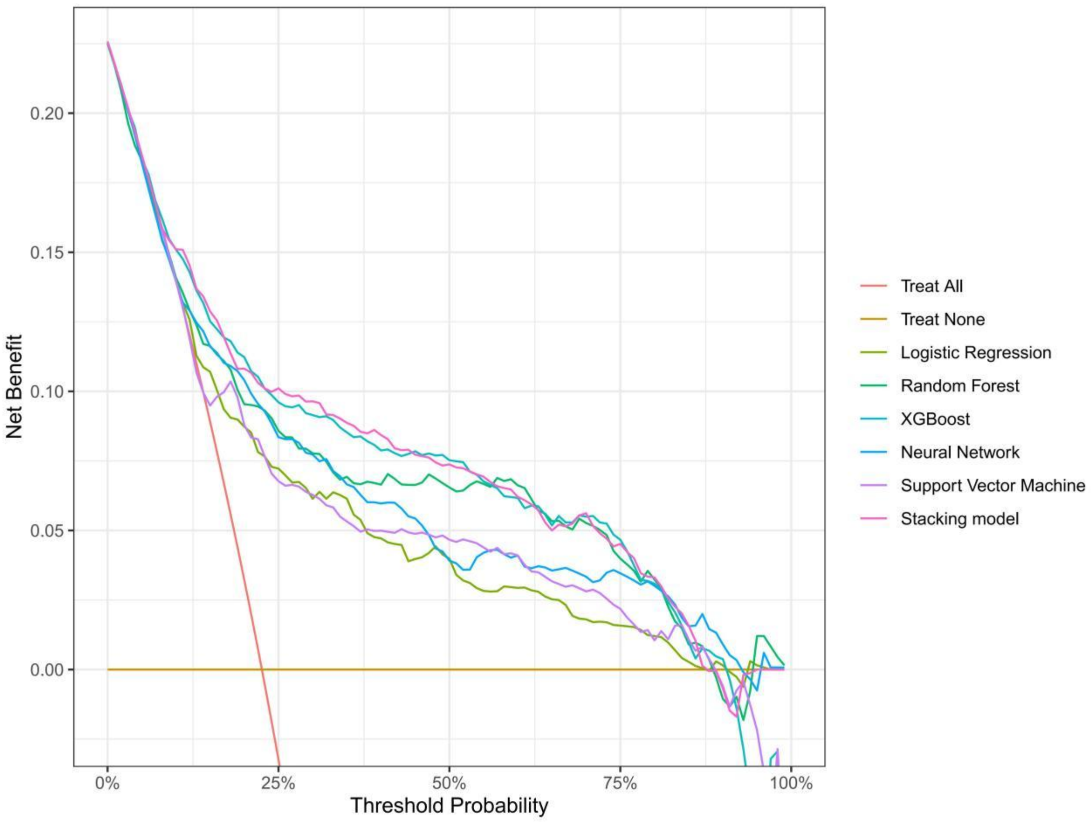

Figure S4. The Decision curves of machine learning predictive models from CMD to CMM.

Table S1. Definition of risk factors in the study [Total].

Risk factors	Notes
Sex	Sex was self-reported and referred to sex assigned at birth.<br>“Male”<br>“Female”
Age	Age was self-reported.<br>“< 60 years old”<br>“≥ 60 years old”
Education	Education was self-reported.<br>“Less than primary”<br>“Primary and above”
Marital status	Marital status was self-reported.<br>“Married” incuded currently married or cohabiting.<br>“Unmarried” inclued never married, separated, divorced, and widowed.
GDP per capita (Yuan)	Division according to the median GDP per capita.<br>“Low” (Capita-level GDP ≤ median GDP per capita 63,399 Yuan)<br>“High” (Capita-level GDP > median GDP per capita 63,399 Yuan)
Unhealthy behavior	Unhealthy behavior is a custom variable, which is the sum of the scores of five conventional factors (smoke status, drink status, physical activity, social status, vegetable intake) . Detailed information is showed in Supplementary Table S2.
Physical fitness impairment	Physical fitness impairment is a custom variable, which is the sum of the scores of four conventional factors (grip strength, pulmonary function, pulse, pre-hypertension). Detailed information is showed in Supplementary Table S3.
IC impairment	IC impairment is a custom variable, which is the sum of the scores of five conventional factors (locomotion, vitality, cognition, depression, sensory). Detailed information is showed in Supplementary Table S4.
$PM_{2.5}$ (μg/m^3)	Averages of $PM_{2.5}$ concentrations from the one year preceding each investigation to the survey date.
Temperature	Averages of temperature concentrations from the one year preceding each investigation to the survey date.
Relative humidity	Averages of relative humidity concentrations from the one year preceding each investigation to the survey date.

Table S2. Unhealthy behavior's definition in the study.

Variable	Notes
Smoking status	Smoking status was self-reported.<br>"Non-smoker" referred to never used smokeless tobacco or smoked tobacco.<br>"Smoker" referred to having ever used smokeless tobacco (manufactured or hand roll cigarettes, cigars, cheroots), smoked tobacco (included chewed, sucked or inhaled) or having ever used tobacco but no more using now.
Drinking status	Drinking status was self-reported.<br>"Non-drinker" referred to never consumed alcoholic beverages (beer, wine, etc.).<br>"Drinker" referred to consuming alcoholic beverages, or having ever consumed but no more consuming now.
Physical activity	Physical activity condition was depended on the vigorous-intensity and moderate-intensity exercise.<br>"low" referred to not doing exercise.<br>"high" referred to having vigorous-intensity or moderate-intensity exercise.
Social engagement	Social engagement was self-reported.(gotten out of the house/your dwelling to attend social meetings, activities, programs or events or to visit friends or relatives)<br>"Unhealthy"included never.<br>"Healthy" included once or twice per year, once or twice per month, once or twice per week, daily.
Vegetable intake	Measured as the number of servings on a typical day[1].<br>"Unhealthy" referred to eating fewer than five servings.<br>"Healthy" referred to eating five or more servings.

Notes: The variance against with guidelines and recommendations were coded as "1", and the "unhealthy behavior" variable was calculated as the sum of five factors, with a total scores ranging from 0 to 5.

Table S3. Physical fitness impairment's definition in the study.

Variance	Notes
Grip strength	Choose the mean of grip strength on the Advantageous hand. Division based on the Asian Working Group for Sarcopenia[2]. "Unhealthy"： Male < 28 kg， Female < 18 kg "Healthy"： Male ≥ 28 kg， Female ≥ 18 kg
Pulmonary Function	Division based on commonly-used qualitative way[3]。 "Unhealthy" referre to (FEV1/FVC) ＜ 70%, "Healthy" referred to (FEV1/FVC) ≥ 70%.
Pulse	The value was calculated based on three measurement. "Unhealthy": < 60 or > 100 beats per minute; "Healthy": 60-100 beats per minute.
Blood Pressure	The average systolic and diastolic blood pressure values were calculated separately based on the three measurements and used as the participant's systolic and diastolic blood pressure. Division based on Clinical practice guidelines for the management of hypertension in China[4]. "Unhealthy": with a systolic blood pressure (SBP) > 120 mmHg or a diastolic blood pressure (DBP) > 80 mmHg. "Healthy": with a systolic blood pressure (SBP) ≤ 120 mmHg and a diastolic blood pressure (DBP) ≤ 80 mmHg.

Notes: The variance against with guidelines and recommendations were coded as "1", and the "Physical fitness impairment" variable was calculated as the sum of four factors, with a total scores ranging from 0 to 4.

Table S4. Definitions of Intrinsic Capacity Measurement Items included in the study

Domain	Question(In the last 30 days), all the responses are in the range of 1 to 5 (none = 1, mild = 2, moderate = 3, severe = 4, extreme/cannot do = 5)
Mobility	how much difficulty did you have with moving around?
Vitality	how much difficulty did you have due to not feeling rested and refreshed during the day (for example, feeling tired, not having energy)?
Cognition	how much difficulty did you have in learning a new task (for example, learning how to get to a new place, learning a new game, learning a new recipe)?
Depression	how much difficulty did you have with feeling sad, low or depressed?
Sensory	How much difficulty did you have in seeing and recognising an object at arm's length (for example, reading)?<br>How much difficulty did you have in hearing and understanding what two people were saying in a quiet room?

Note: The score of domain mora than 1 point were coded as "1", and the "IC impairment" variable was calculated as the sum of five domains, with a total scores ranging from 0 to 5.

Table S5. Indicators of predictive different models from Health to CMD

ML models	AUC	Accuracy	Sensitivity	Specificity	Brier Score	Calibration Slope	Calibration Intercept
XGBoost	0.89 (95%CI: 0.88-0.91)	0.84 (95%CI: 0.82-0.86)	0.71	0.99	0.12	0.93	-0.09
RF	0.89 (95%CI: 0.87-0.90)	0.84 (95%CI: 0.82-0.86)	0.72	0.98	0.12	0.62	-0.15
LR	0.80 (95%CI: 0.78-0.82)	0.74 (95%CI: 0.71-0.76)	0.70	0.77	0.18	0.93	0.08
SVM	0.84 (95%CI: 0.82-0.85)	0.77 (95%CI: 0.74-0.79)	0.68	0.86	0.16	0.95	0.05
NN	0.88 (95%CI: 0.86-0.90)	0.82 (95%CI: 0.80-0.84)	0.73	0.91	0.13	0.89	-0.06
Stacking model	0.89 (95%CI: 0.88-0.91)	0.85 (95%CI: 0.83-0.87)	0.74	0.98	0.12	0.93	0.02

Table S6. Indicators of predictive different models from CMD to CMM

ML models	AUC	Accuracy	Sensitivity	Specificity	Brier Score	Calibration Slope	Calibration Intercept
XGBoost	0.74 (95%CI: 0.68-0.79)	0.86 (95%CI: 0.83-0.88)	0.50	0.93	0.12	0.83	-0.25
RF	0.74 (95%CI: 0.69-0.80)	0.82 (95%CI: 0.79-0.85)	0.51	0.89	0.13	0.69	-0.41
LR	0.73 (95%CI: 0.68-0.78)	0.77 (95%CI: 0.74-0.80)	0.56	0.82	0.14	1.03	-0.06
SVM	0.68 (95%CI: 0.62-0.74)	0.83 (95%CI: 0.80-0.86)	0.46	0.90	0.14	0.88	-0.24
NN	0.72 (95%CI: 0.66-0.77)	0.78 (95%CI: 0.75-0.81)	0.50	0.83	0.13	0.88	-0.22
Stacking model	0.76 (95%CI: 0.72-0.82)	0.76 (95%CI: 0.73-0.79)	0.65	0.78	0.12	1.05	-0.02

Table S7. Hazard ratios (HRs) and 95% confidence intervals (CIs) for each transition in CMM progression among participants with 2 year environmental exposures.

Group	Health to CMD		CMD to CMM	
	HR (95%CI)	*P*	HR (95%CI)	*P*
Sex	1.14 (95%CI: 1.06-1.22)	<0.001	1.23(95%CI: 1.03-1.46)	0.020
Age	1.16 (95%CI: 1.09-1.23)	<0.001	0.98(95%CI: 0.84-1.14)	0.799
Marital status	1.03 (95%CI: 0.94-1.12)	0.526	1.10(95%CI: 0.90-1.35)	0.340
Education	0.91 (95%CI: 0.86-0.97)	0.003	1.07(95%CI: 0.91-1.25)	0.412
GDP per capita	0.74 (95%CI: 0.70-0.79)	<0.001	0.87(95%CI: 0.74-1.02)	0.087
IC impairment	1.00 (95%CI: 0.97-1.03)	0.756	1.17(95%CI: 1.09-1.27)	<0.001
Unhealthy behavior	1.08 (95%CI: 1.03-1.13)	<0.001	1.09(95%CI: 0.98-1.22)	0.101
Physical fitness impairment	1.17 (95%CI: 1.11-1.24)	<0.001	1.10(95%CI: 0.96-1.27)	0.171
$PM_{2.5}$	1.04 (95%CI: 1.03-1.05)	<0.001	1.06(95%CI: 1.05-1.07)	<0.001
Relative Humidity	0.98 (95%CI: 0.97-0.99)	0.035	0.97(95%CI: 0.96-0.98)	<0.001
Temperature	0.97 (95%CI: 0.96-0.98)	<0.001	0.94(95%CI: 0.91-0.97)	<0.001

Notes: Sex, ref: male; Age,ref: < 60 (years); Marital status, ref: married; Education, ref: less than primary; GDP per capita, ref: Low; $PM_{2.5}$ (per 1 $\mu g/m^3$)

Table S8. Hazard ratios (HRs) and 95% confidence intervals (CIs) for each transition in CMM progression among participants with 3 year environmental exposures.

Group	Health to CMD		CMD to CMM	
	HR(95%CI)	*P*	HR(95%CI)	*P*
Sex	1.14 (95%CI: 1.06-1.22)	<0.001	1.23 (95%CI: 1.03-1.46)	0.021
Age	1.16 (95%CI: 1.09-1.23)	<0.001	0.99 (95%CI: 0.84-1.15)	0.869
Marital status	1.03 (95%CI: 0.94-1.12)	0.550	1.11 (95%CI: 0.90-1.36)	0.318
Education	0.91 (95%CI: 0.86-0.97)	0.003	1.08 (95%CI: 0.92-1.26)	0.362
GDP per capita	0.75 (95%CI: 0.70-0.80)	<0.001	0.94 (95%CI: 0.80-1.10)	0.422
IC impairment	1.00 (95%CI: 0.97-1.03)	0.803	1.17 (95%CI: 1.08-1.26)	<0.001
Unhealthy behavior	1.08 (95%CI: 1.03-1.13)	0.001	1.08 (95%CI: 0.97-1.20)	0.164
Physical fitness impairment	1.18 (95%CI: 1.12-1.25)	<0.001	1.12 (95%CI: 0.97-1.28)	0.123
$PM_{2.5}$	1.03 (95%CI: 1.02-1.04)	<0.001	1.05 (95%CI: 1.04-1.06)	<0.001
Relative Humidity	0.98 (95%CI: 0.97-0.99)	0.011	0.98 (95%CI: 0.97-0.99)	<0.001
Temperature	0.97 (95%CI: 0.96-0.98)	<0.001	0.94 (95%CI: 0.91-0.97)	<0.001

Notes: Sex, ref: male; Age,ref: < 60 (years); Marital status, ref: married; Education, ref: less than primary; GDP per capita, ref: Low; $PM_{2.5}$ (per 1 μg/m^3)

Table S9. Hazard ratios (HRs) and 95% confidence intervals (CIs) for each transition in CMM progression by Cox regression.

Group	Health to CMD		CMD to CMM	
	HR(95%CI)	*P*	HR(95%CI)	*P*
Sex	1.14 (95%CI: 1.06-1.22)	<0.001	1.33 (95%CI: 1.12-1.59)	0.002
Age	1.16 (95%CI: 1.09-1.23)	<0.001	0.94 (95%CI: 0.80-1.10)	0.415
Marital status	1.02 (95%CI: 0.93-1.11)	0.718	0.97 (95%CI: 0.79-1.20)	0.813
Education	0.90 (95%CI: 0.84-0.96)	<0.001	1.18 (95%CI: 1.01-1.39)	0.042
GDP per capita	0.68 (95%CI: 0.64-0.72)	<0.001	0.82 (95%CI: 0.70-0.96)	0.011
IC impairment	0.99 (95%CI: 0.96-1.02)	0.653	1.11 (95%CI: 1.03-1.20)	0.007
Unhealthy behavior	1.09 (95%CI: 1.04-1.13)	<0.001	1.16 (95%CI: 1.04-1.29)	0.009
Physical fitness impairment	1.16 (95%CI: 1.10-1.23)	<0.001	1.16 (95%CI: 1.01-1.34)	0.041
$PM_{2.5}$	1.05 (95%CI: 1.04-1.06)	<0.001	1.07 (95%CI: 1.06-1.08)	<0.001
Relative Humidity	1.00 (95%CI: 0.99-1.01)	0.456	1.03 (95%CI: 1.01-1.06)	0.005
Temperature	0.95 (95%CI: 0.94-0.96)	<0.001	0.92 (95%CI: 0.89-0.96)	<0.001

Notes: Sex, ref: male; Age,ref: < 60 (years); Marital status, ref: married; Education, ref: less than primary; GDP per capita, ref: Low; $PM_{2.5}$ (per 1 $\mu g/m^3$)

Table S10. Basic information of the study participants in CMM progression without baseline chronic conditions.

Group	Number (%) / Mean±sd		
	Health	CMD	CMM
**Total**	4,193 (100.00%)	2,169 (51.73%)	327 (15.07%)
**Sex**			
Male	2,193 (52.30%)	1,153 (53.16%)	162 (49.54%)
Female	2,000 (47.70%)	1,016 (46.84%)	165 (50.46%)
**Age (year)**			
<60	2,331 (55.59%)	390 (17.98%)	44 (13.46%)
≥60	1,862 (44.41%)	1,779 (82.02%)	283 (86.54%)
**Marital status**			
Married	3,693 (88.08%)	1,873 (86.35%)	279 (85.32%)
Unmarried	500 (11.92%)	296 (13.65%)	48 (14.68%)
**Education**			
Less than primary	1323 (31.55%)	760 (35.04%)	100 (30.58%)
Primary and above	2,870 (68.45%)	1,409 (64.96%)	227 (69.42%)
**GDP (Yuan)**			
High	1,943 (46.34%)	994 (45.83%)	180 (55.05%)
Low	2,250 (53.66%)	1,175 (54.17%)	147 (44.95%)
**IC impairment**	1.67±1.32	2.03±1.44	2.45±1.38
**unhealthy behavior**	2.00±1.03	2.70±1.10	2.67±1.13
**physical fitness impairment**	1.33±0.75	1.50±0.70	1.56±0.76

Note: IC impairment was calculated based on mobility, vitality, cognition, depression, and sensory function (range 0-5). Unhealthy behavior was calculated based on smoking, drinking, physical activity, social isolation, and low vegetable intake (range 0-5). Physical fitness impairment was calculated based on grip strength, lung capacity, pulse rate, and prehypertension (range 0-4). Details are provided in Tables S2-S4.

Table S11. Hazard ratios (HRs) and 95% confidence intervals (CIs) for each transition in CMM progression among participants without baseline chronic conditions.

Group	Health to CMD		CMD to CMM	
	HR (95%CI)	*P*	HR (95%CI)	*P*
Sex	1.10 (95%CI: 1.03-1.19)	0.008	1.15 (95%CI: 0.96-1.38)	0.075
Age	1.14 (95%CI: 1.07-1.21)	<0.001	0.95 (95%CI: 0.80-1.11)	0.503
Marital status	1.03 (95%CI: 0.94-1.13)	0.481	1.12 (95%CI: 0.90-1.39)	0.295
Education	0.91 (95%CI: 0.85-0.97)	0.004	1.08 (95%CI: 0.91-1.28)	0.386
GDP per capita	0.75 (95%CI: 0.70-0.80)	0.000	0.79 (95%CI: 0.67-0.93)	0.006
IC impairment	1.00 (95%CI: 0.97-1.03)	0.810	1.18 (95%CI: 1.09-1.28)	<0.001
Unhealthy behavior	1.06 (95%CI: 1.01-1.11)	0.009	1.03 (95%CI: 0.92-1.15)	0.605
Physical fitness impairment	1.16 (95%CI: 1.10-1.23)	<0.001	1.05 (95%CI: 0.90-1.22)	0.532
$PM_{2.5}$	1.04 (95%CI: 1.02-1.04)	<0.001	1.08 (95%CI: 1.07-1.09)	<0.001
Relative Humidity	1.00 (95%CI: 0.99-1.01)	0.254	0.97 (95%CI: 0.96-0.98)	<0.001
Temperature	0.96 (95%CI: 0.95-0.98)	<0.001	0.93 (95%CI: 0.90-0.96)	<0.001

Ref: Sex (male), Age (<60 years), Education (less than primary), GDP per capita (Low), $PM_{2.5}$ (per 1 $\mu g/m^3$).

Table S12. Indicators of predictive different models from Health to CMD without baseline diseases

ML models	AUC	Accuracy	Sensitivity	Specificity	Brier Score	Calibration Slope	Calibration Intercept
XGBoost	0.90 (95%CI: 0.88-0.91)	0.84 (95%CI: 0.82-0.86)	0.72	0.98	0.11	0.98	-0.01
RF	0.90 (95%CI: 0.88-0.92)	0.84 (95%CI: 0.82-0.86)	0.74	0.96	0.11	0.90	-0.09
LR	0.81 (95%CI: 0.78-0.83)	0.75 (95%CI: 0.72-0.77)	0.73	0.76	0.17	1.14	-0.01
SVM	0.84 (95%CI: 0.83-0.87)	0.78 (95%CI: 0.76-0.80)	0.75	0.82	0.15	1.11	-0.02
NN	0.85 (95%CI: 0.83-0.87)	0.79 (95%CI: 0.77-0.82)	0.74	0.86	0.16	0.98	0.02
Stacking model	0.90 (95%CI: 0.89-0.92)	0.85 (95%CI: 0.83-0.86)	0.74	0.97	0.10	1.10	0.01

Table S13. Indicators of predictive different models from CMD to CMM without baseline diseases

ML models	AUC	Accuracy	Sensitivity	Specificity	Brier Score	Calibration Slope	Calibration Intercept
XGBoost	0.74 (95%CI: 0.68-0.81)	0.88 (95%CI: 0.86-0.91)	0.47	0.95	0.12	0.57	-0.46
RF	0.76 (95%CI: 0.70-0.81)	0.84 (95%CI: 0.82-0.86)	0.54	0.89	0.12	0.47	-0.71
LR	0.72 (95%CI: 0.67-0.78)	0.78 (95%CI: 0.75-0.81)	0.56	0.81	0.12	1.05	0.26
SVM	0.70 (95%CI: 0.63-0.76)	0.80 (95%CI: 0.76-0.83)	0.74	0.84	0.13	1.02	0.16
NN	0.75 (95%CI: 0.69-0.81)	0.68 (95%CI: 0.65-0.72)	0.71	0.68	0.12	0.65	-0.42
Stacking model	0.77 (95%CI: 0.72-0.83)	0.77 (95%CI: 0.73-0.80)	0.68	0.77	0.11	0.98	0.06

Table S14. Hazard ratios (HRs) and 95% confidence intervals (CIs) for each transition in CMM progression among participants based on a 70% Random subsample.

Group	Health to CMD		CMD to CMM	
	HR (95%CI)	*P*	HR (95%CI)	*P*
Sex	1.13 (95%CI: 1.03-1.23)	0.007	1.24 (95%CI: 1.01-1.52)	0.045
Age	1.15 (95%CI: 1.06-1.23)	<0.001	1.05 (95%CI: 0.87-1.26)	0.613
Marital status	1.03 (95%CI: 0.93-1.14)	0.564	1.02 (95%CI: 0.80-1.29)	0.894
Education	0.91 (95%CI: 0.84-0.98)	0.012	1.05 (95%CI: 0.87-1.27)	0.602
GDP per capita	0.75 (95%CI: 0.70-0.81)	<0.001	0.76 (95%CI: 0.62-0.92)	0.005
IC impairment	0.98 (95%CI: 0.95-1.02)	0.359	1.10 (95%CI: 1.01-1.21)	0.035
Unhealthy behavior	1.07 (95%CI: 1.02-1.13)	0.009	1.09 (95%CI: 0.95-1.24)	0.205
Physical fitness impairment	1.16 (95%CI: 1.08-1.23)	<0.001	0.99 (95%CI: 0.84-1.16)	0.877
$PM_{2.5}$	1.04 (95%CI: 1.03-1.05)	<0.001	1.08 (95%CI: 1.07-1.09)	<0.001
Relative Humidity	0.98 (95%CI: 0.99-1.01)	0.085	0.97 (95%CI: 0.96-0.98)	<0.001
Temperature	0.96 (95%CI: 0.94-0.97)	<0.001	0.92 (95%CI: 0.89-0.95)	<0.001

Ref: Sex (male), Age (<60 years), Education (less than primary), GDP per capita (Low), $PM_{2.5}$ (per 1 $\mu g/m^3$).

Table S15. Hazard ratios (HRs) and 95% confidence intervals (CIs) for each transition in CMM progression (obesity: BMI≥28 kg/m^2)

Group	Health to CMD		CMD to CMM	
	HR (95%CI)	*P*	HR (95%CI)	*P*
Sex	1.11 (95%CI: 1.04-1.20)	0.003	1.24 (95%CI: 1.05-1.47)	0.011
Age	1.14 (95%CI: 1.07-1.22)	<0.001	0.95 (95%CI: 0.82-1.11)	0.529
Marital status	1.02 (95%CI: 0.93-1.11)	0.670	1.07 (95%CI: 0.88-1.30)	0.482
Education	0.93 (95%CI: 0.87-0.99)	0.017	1.01 (95%CI: 0.87-1.18)	0.884
GDP per capita	0.75 (95%CI: 0.71-0.80)	<0.001	0.67 (95%CI: 0.57-0.78)	<0.001
IC impairment	0.99 (95%CI: 0.96-1.02)	0.611	1.10 (95%CI: 1.02-1.18)	0.014
Unhealthy behavior	1.08 (95%CI: 1.03-1.12)	0.001	1.12 (95%CI: 1.01-1.24)	0.030
Physical fitness impairment	1.14 (95%CI: 1.08-1.21)	<0.001	0.99(95%CI: 0.86-1.13)	0.828
$PM_{2.5}$	1.04 (95%CI: 1.03-1.05)	<0.001	1.09 (95%CI: 1.08-1.10)	<0.001
Relative Humidity	1.00 (95%CI: 0.99-1.01)	0.302	0.97 (95%CI: 0.96-0.98)	<0.001
Temperature	0.96 (95%CI: 0.95-0.97)	<0.001	0.93 (95%CI: 0.90-0.96)	<0.001

Ref: Sex (male), Age (<60 years), Education (less than primary), GDP per capita (Low), $PM_{2.5}$ (per 1 μg/m^3).

Table S16. Indicators of predictive different models from Health to CMD (obesity: BMI≥28 kg/m^2)

ML models	AUC	Accuracy	Sensitivity	Specificity	Brier Score	Calibration Slope	Calibration Intercept
XGBoost	0.89 (95%CI:0.87-0.91)	0.84 (95%CI: 0.82-0.86)	0.73	0.97	0.12	0.78	-0.08
RF	0.89 (95%CI:0.87-0.91)	0.84 (95%CI: 0.82-0.86)	0.73	0.98	0.12	0.76	-0.11
LR	0.80 (95%CI:0.77-0.82)	0.74 (95%CI: 0.71-0.76)	0.72	0.76	0.18	0.93	0.07
SVM	0.83 (95%CI:0.81-0.86)	0.77 (95%CI: 0.74-0.79)	0.69	0.86	0.16	0.96	0.05
NN	0.84 (95%CI:0.83-0.87)	0.78 (95%CI: 0.76-0.80)	0.65	0.93	0.18	0.77	0.01
Stacking model	0.90 (95%CI:0.88-0.91)	0.84 (95%CI: 0.81-0.86)	0.73	0.97	0.12	0.99	-0.01

Table S17. Indicators of predictive different models from CMD to CMM (obesity: BMI≥28 kg/m^2)

ML models	AUC	Accuracy	Sensitivity	Specificity	Brier Score	Calibration Slope	Calibration Intercept
XGBoost	0.76 (95%CI: 0.72-0.82)	0.84 (95%CI: 0.81-0.86)	0.60	0.85	0.13	0.76	-0.10
RF	0.76 (95%CI: 0.72-0.81)	0.86 (95%CI: 0.83-0.88)	0.58	0.84	0.13	0.74	-0.15
LR	0.69 (95%CI: 0.64-0.74)	0.67 (95%CI: 0.64-0.71)	0.55	0.78	0.16	0.94	0.02
SVM	0.68 (95%CI: 0.62-0.73)	0.72 (95%CI: 0.69-0.76)	0.59	0.73	0.15	1.03	0.11
NN	0.71 (95%CI: 0.66-0.76)	0.80 (95%CI: 0.77-0.83)	0.45	0.88	0.14	0.82	0.12
Stacking model	0.77 (95%CI: 0.72-0.82)	0.86 (95%CI: 0.82-0.88)	0.64	0.82	0.13	0.97	0.10

Reference

1.Wu F, Guo Y, Chatterji S, et al. Common risk factors for chronic non-communicable diseases among older adults in China, Ghana, Mexico, India, Russia and South Africa: the study on global AGEing and adult health (SAGE) wave 1.BMC Public Health. 2015;15:88. Published 2015 Feb 6. doi:10.1186/s12889-015-1407-0

2. Chen LK, Woo J, Assantachai P, et al. Asian Working Group for Sarcopenia: 2019 Consensus Update on Sarcopenia Diagnosis and Treatment. J Am Med Dir Assoc. 2020;21(3):300-307.e2. doi:10.1016/j.jamda.2019.12.012

3. Ponce MC, Sankari A, Sharma S. Pulmonary Function Tests. [Updated 2023 Aug 28]. In: StatPearls [Internet]. Treasure Island (FL): StatPearls Publishing; 2025 Jan-. Available from: https://www.ncbi.nlm.nih.gov/books/NBK482339/#

4. Chinese Hypertension Prevention and Treatment Guidelines Revision Committee, Hypertension Alliance (China), Hypertension Branch of China International Exchange and Promotion Association for Medical and Health Care, et al. Chinese Guidelines for the Prevention and Treatment of Hypertension (2024 Revised Edition) [J]. China J Hypertens, 2024, 32(07): 603-700.
